# Cerebellar Compression by Giant Extracanalicular Osteoma with Central Cholesterol Granuloma

**DOI:** 10.1155/2023/6652012

**Published:** 2023-12-15

**Authors:** Caroline F. Smith, Conner J. Massey, Scott E. Mann

**Affiliations:** ^1^University of Colorado School of Medicine, Aurora, Colorado, USA; ^2^University of Colorado School of Medicine, Department of Otolaryngology Head and Neck Surgery, Aurora, Colorado, USA; ^3^Denver Health Medical Center, Department of Surgery, Trauma, Denver, CO, USA

## Abstract

Temporal bone osteomas comprise 0.1–1% of benign tumors involving the skull, the majority of which arise in the external auditory canal. More rarely, they can arise from the mastoid portion of the temporal bone. These generally present as a slow growing skull base lesion that can cause cosmetic deformity, headache, and/or hearing loss. Here, we report a case of extracanalicular mastoid osteoma uniquely presenting with posterior fossa and cerebellar compression with associated dizziness and imbalance.

## 1. Introduction

The etiology of extracanalicular mastoid osteomas is unknown. They may occur in isolation due to trauma, chronic infection, irradiation, or as a part of a syndrome. As many of these tumors present in young women, some theorize that changes in the hormonal milieu during young adulthood are linked to their development [1–3]. Noncontrast computed tomography (CT) of the temporal bone is key in diagnosis and often demonstrates bony, exophytic, and osseous lesions [2]. Diagnosis is confirmed by histopathology showing either dense sclerotic bone (compact type), mature bone with a marrow system (spongy type), or a mixed type [3]. The prognosis of mastoid osteoma is generally excellent, and recurrence is uncommon [1, 2].

## 2. Case Presentation

**Figure 1:**
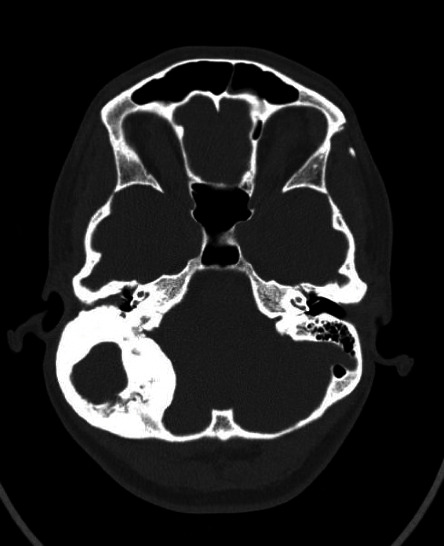
Initial computed tomography scan showing right bony thickening, sclerosis, and expansion centered around right posterior petrous temporal bone with extension into the posterior external auditory canal with central lucent structure. There is compression of the right posterior fossa.

A 24-year-old female presented to the emergency department with an intermittent yet progressive headache, dizziness, and hearing loss with aural pressure. These symptoms began in childhood and had worsened over the prior four years. As her dizziness worsened, she grew unable to work and quit her job. In the emergency department, the provider noted obstruction of the right external auditory canal (EAC). Wick placement was attempted but was met with bony resistance. A CT head demonstrated an expansile bony mass of the right posterior temporal bone measuring 4.7 × 5.0 × 5.7 cm with a central fluid-filled cavity (Figure 1). When questioned about this, the patient reported that this mass had been present since childhood and it had been gradually increasing in size.

At the patient's initial ENT clinic visit, physical exam was significant for bony stenosis of the right EAC with an obvious asymmetric deformity of the right mastoid and occipital skull. Pure tone audiogram (PTA) showed moderately severe rising to moderate conductive hearing loss in the right ear with normal thresholds of the left ear.

**Figure 2:**
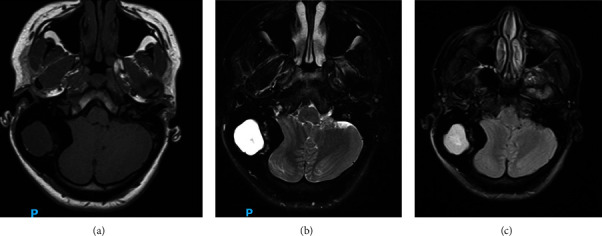
Magnetic resonance imaging in T1 (a), T2 (b), and T2 flair (c) sequencing showing bony expansion and sclerosis of the right temporal bone, fluid within this expansion, and mass effect involving the posterior fossa.

An MRI was obtained (Figure 2) which demonstrated significant cerebellar compression and tonsillar herniation through the foramen magnum; a lack of cerebral edema indicated that this was likely a chronic finding. Neurosurgery was consulted and they recommended confirmation of diagnosis prior to any intracranial surgical intervention.

The patient was taken to the OR for an open biopsy (cortical mastoidectomy) and canalplasty. Intraoperatively, the mass was composed of extremely dense bone, as well as a central compartment filled with thick, dark fluid and minimal soft tissue. Biopsy specimens were sent for both the dense bone of the mass and the soft tissue within the central cavity. The bone obstructing the EAC was then removed with an otologic drill and curette, revealing a medial EAC filled with keratinaceous debris. The tympanic membrane was intact and the middle ear was well aerated. A skin split thickness skin graft was used to complete the EAC reconstruction. Pathology of the bony fragments showed benign, disorderly proliferation of compact bone, consistent with osteoma. Pathology of the central compartment showed fibroconnective tissue with cholesterol granuloma.

**Figure 3:**
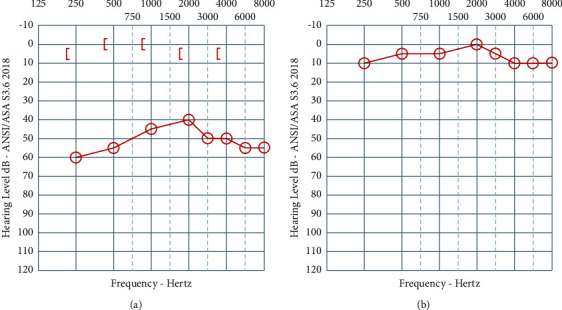
Preoperative audiometric evaluation (a) showing right moderate conductive hearing loss. Postoperative audiometric evaluation (b) showing normal right hearing thresholds.

Postoperatively, the patient's hearing improved (Figure 3) and testing revealed normal hearing thresholds in the right ear. In addition, the patient's headaches improved substantially, though the subjective imbalance persisted. Her symptom improvement was enough that she returned to work and elected against any further intervention. After one year, she was lost to follow up to the ENT service, though in subsequent years she was also diagnosed with major depressive disorder, migraine disorder, and PTSD and began treatment for those conditions.

Five years after her surgery, she presented to the ED with an altered mental status. A CT head without contrast was obtained and demonstrated a stable appearance of the osteoma, measuring 5.1 × 4.9 × 5.4 cm. The mass effect on the posterior fossa remained unchanged. Neurologic exam was unremarkable, and the patient's acute symptoms were attributed to a change in her psychiatric medications. The patient was subsequently seen in the ENT clinic, and her exam was unchanged from the 5 years prior. Her EAC remained patent, and repeat testing revealed normal hearing. While she did continue to describe intermittent headaches, the patient reported benefit from migraine treatment and that her headaches were manageable. Though her balance was still subjectively poor, it was not interfering with her daily life, and her severe dizziness remained resolved. No further surgical interventions are currently planned.

## 3. Discussion

Extracanalicular mastoid osteomas represent a rare, benign tumor of the skull. Initial differential diagnosis for bony tumors of the mastoid includes osteoma, Paget's disease, osteosarcoma, monostotic fibrous dysplasia, multiple myeloma, giant cell tumor, and osteoblastic metastasis [2]. The most common presentation of extracanalicular mastoid osteomas includes hearing loss, cosmetic concerns, and headache. However, intracranial compression from mastoid osteomas is rare. There are reports of intracranial compression involving the facial nerve and sigmoid sinus, but no reported cases of posterior fossa compression as exhibited in this case [4].

We describe a unique presentation of extracanalicular mastoid osteoma involving substantial intracranial compression in the posterior fossa. To our knowledge, this is the only existing documented case of mastoid osteoma causing posterior fossa compression. In addition, an osteoma with a central compartment containing cholesterol granuloma is unique in the literature. There have been numerous case reports of large mastoid cholesterol granuloma, but none in association with osteoma [5].

Extracanalicular mastoid osteomas are typically managed with surgical resection only when symptomatic or causing cosmetic deformities [2]. We opted initially for a symptom-directed surgery to address her conductive hearing loss and relieve pressure of the stenotic EAC while confirming the diagnosis via biopsy prior to a more morbid intracranial resection.

While surgical intervention did not change her cerebellar compression, canalplasty did reduce the pressure of her stenotic EAC and removed the medial accumulation of keratinaceous debris. We postulate that the patient's known migraine disorder was also contributing to her severe headaches and dizziness, and the EAC stenosis and accumulating debris were acting as triggers. The relief of these stimuli led to improvements in both pain and dizziness despite the lack of intervention on the cerebellar compression. Though her imbalance continued, her episodic dizziness and headaches were improved enough to elect against further surgical intervention. The definitive resection for this tumor would be quite morbid in this young patient. Given her stable symptomatic improvement for more than five years, there are no current plans for further intervention.
